# Exploring the gap: attitudes, knowledge, and training needs in complementary and integrative medicine among healthcare professionals at German university hospitals

**DOI:** 10.3389/fmed.2024.1408653

**Published:** 2024-05-09

**Authors:** Daniela Hesmert, Carina Klocke, Regina Stolz, Roman Huber, Yvonne Samstag, Katrin Hübner, Thomas Simmet, Tatiana Syrovets, Stefanie Joos, Jan Valentini

**Affiliations:** ^1^Institute of General Practice and Interprofessional Care, University Hospital and Faculty of Medicine, Tübingen, Germany; ^2^Center for Complementary Medicine, Department of Medicine II, Medical Center, Faculty of Medicine – University of Freiburg, Freiburg im Breisgau, Germany; ^3^Section of Molecular Immunology, Institute of Immunology, Heidelberg University Hospital, Heidelberg, Germany; ^4^Institute of Experimental and Clinical Pharmacology, Toxicology, and Pharmacology of Natural Products, Ulm University, Ulm, Germany

**Keywords:** complementary medicine, integrative medicine, healthcare professional, attitude, knowledge, needs, university hospital, Germany

## Abstract

**Introduction:**

The use of Complementary and Integrative Medicine (CIM) is very popular among the general population in Germany. However, international studies show that nurses, physicians, and other health care professionals (HCPs) at hospitals often do not feel sufficiently informed about different CIM approaches. Moreover, they do not feel trained enough to counsel their patients appropriately. In the German-speaking context, particularly within university hospitals, research on this subject is scarce. Therefore, the aim of this explorative study was to evaluate attitudes, subjective knowledge, and needs regarding CIM among HCPs with direct patient interaction across all four university hospitals in the federal state of Baden-Württemberg, Germany (Tübingen, Ulm, Freiburg, Heidelberg).

**Methods:**

The multicenter, cross-sectional, anonymous full survey was conducted online using a self-developed, semi-structured, web-based questionnaire. Recruitment took place via all-inclusive e-mail distribution lists of all four university hospitals.

**Results:**

A total of *n* = 2,026 participants (response rate varied by location from about 5 to 14%) fully answered the questionnaire. Nurses constituted the largest professional group (*n* = 1,196; 59%), followed by physicians (*n* = 567; 28%), physiotherapists (*n* = 54), psychologists (*n* = 48), midwives (*n* = 37), and other professions (*n* = 124). More than two-thirds (71%, *n* = 1,437) of the participants were female and 14% (*n* = 286) reported additional training in CIM. The overall attitude toward CIM (10-point Likert scale, 10 = “very favorable”) was clearly positive (*M* ± *SD*: 7.43 ± 2.33), with notable differences between professional groups: midwives (9.05 ± 1.18), physiotherapists (8.44 ± 1.74), and nurses (8.08 ± 1.95) expressed the highest support, whereas physicians (5.80 ± 2.39) the lowest. 42% of the participants incorporated CIM in patient care (from 33% of physicians to 86% of midwives). Overall, relaxation therapy (*n* = 1,951; 96%), external applications (*n* = 1,911; 94%), massage (*n* = 1,836; 91%), and meditation/mindfulness (*n* = 1,812; 89%) were rated as useful or rather useful for patients. The average self-assessed knowledge level about CIM was moderate (M ± SD: 5.83 ± 2.03). Most of the participants found CIM training at university hospitals important and saw research about CIM as one of the tasks of university hospitals. The participants expressed the highest interest in education for acupuncture/acupressure, relaxation therapies, and manual medicine.

**Discussion:**

This comprehensive survey of health care professionals (HCPs) at university hospitals in Germany reveals a clearly positive disposition toward CIM, aligning with findings from other hospital-based surveys and highlighting differences among professional groups. While most therapies deemed beneficial for patient care are supported by positive evidence, further research is required for others. Given the average self-reported knowledge of CIM, targeted education is essential to meet the needs of both HCPs and patients and to ensure the provision of evidence-based information on the risks and benefits of CIM.

## 1 Introduction

Complementary and integrative medical (CIM) approaches encompass a wide range of methods including nutritional, psychological, and physical approaches (1). While some CIM approaches are recommended in guidelines for health care professionals (HCPs) (2) and show positive results in studies, such as acupuncture (3–5) or relaxation therapy (6) to reduce pain or phytotherapy to prevent urinary infections (7) or cognitive impairment (8) or to reduce chronic constipation (9), others may cause interactions with conventional drugs, such as chemotherapy, and be potentially harmful for patients’ health, such as certain vitamins (10) or diets (11). Also, financial risks due to high costs are possible (12).

Although there is an ongoing discussion on the definition of which CIM therapies fall under the umbrella concept, the terms ‘complementary’, ‘alternative’, and ‘integrative’ are constantly evolving, as described by the National Institutes of Health (1). A recent definition was suggested by Brinkhaus and Esch as follows: *“Integrative medicine affirms the importance of the doctor-patient relationship, aims at the whole person, is informed by evidence and uses all appropriate therapeutic, preventive, health-promoting or lifestyle approaches as well as all disciplines of health care to achieve optimal health and healing-emphasizing both the art and science of healing”* (13). In academic and scientific contexts, authors often have used the abbreviation CAM (“complementary and alternative medicine”) in the past. In recent years, the term “Integrative Medicine” (IM) or “Complementary and Integrative Health” (CIH) is used to describe an evidence-based approach to implementing these therapies in healthcare (1). For this reason, this study uses the term “complementary and integrative medicine” (CIM), even when referring to studies using CAM or IM as concepts.

There is adequate research available on patients’ interest in and demand for CIM. Patients may experience positive effects like resource activation through an improved sense of coherence or patient activation (14) and their efforts toward greater psychological or physical well-being (12, 15). In general, CIM is used for various counseling occasions, for example, by more than one third of patients with cardiovascular diseases (16) and by more than 40% of patients with chronic pain (17). According to international studies, between 32% (18) and 40% (19) of patients in Germany used CIM in the previous 12 months. For cancer patients, a review reported usage by 50% of patients (20) while for some population groups, such as patients with breast cancer, a usage by up to 80% of patients can be assumed (21). Accordingly, recent studies indicate that between 15% (22) and 74% (23) of oncology patients in Germany use CIM during their therapy. Interest in CIM is also high among patients at university hospitals. As we have shown previously, in Baden-Württemberg in Germany patients from different departments of university hospitals had an average usage rate of CIM of 48% for their current disease and 48% asked for counseling on CIM (15). At university hospitals, HCPs such as nurses, physicians, and physiotherapists, are possible points of contact for patients regarding CIM.

According to an international review article, about two-thirds of *nurses* have positive attitudes toward CIM (24). Although more than two-thirds of nurses in Australia discuss this topic with their patients (25) and 50% of nurses report professional use of CIM (26), they also cite a lack of knowledge as a barrier to proper communication about the topic (27). According to quantitative international studies more than two-thirds of nurses report a lack of knowledge about CIM (24). Looking at the attitudes of *physicians*, studies show that they are usually more skeptical about CIM than nurses (28, 29). Like the nurses, only 23% of physicians in a study at a university hospital in Germany considered themselves to be adequately informed (28). Approximately 20% (30) to 60% (31) of general practitioners use CIM in an outpatient setting. Although physicians may be an important source of information on CIM for patients in general (15, 22), there is a lack of evidence toward attitudes and knowledge about CIM procedures for physicians at university hospitals in Germany. As for *other HCPs,* midwives are most likely to support complementary therapies (32, 33). Little is known for other HCPs, such as physiotherapists. Looking at all HCPs in hospitals, a study from an academic center in Switzerland showed that 80% of the different professionals do not feel sufficiently informed about CIM (34). Commonly, female hospital staff show a significantly higher level of interest in CIM than male staff (28). However, there is a general lack of research about HCPs and CIM at university hospitals.

Within the framework of evidence-based medicine, which includes patient’s views, external evidence and professionals’ expertise (35), it is important to know about the attitudes, knowledge and needs of HCPs. As shown, there are several studies concerning several HCP groups in different settings. However, the setting of university hospitals in the German-speaking area has not been thoroughly investigated. Therefore, the aim of this study was to assess the attitudes toward CIM and ratings of specific CIM therapies among different HCP groups at university hospitals in Baden-Württemberg, Germany. In addition, we aimed to investigate HCPs’ usage of CIM, their self-assessed level of knowledge about CIM and their interest in training in specific CIM procedures.

## 2 Materials and methods

### 2.1 Study design

We conducted an anonymous, multicenter, cross-sectional full survey using a self-administered, semi-structured online questionnaire.

The study was registered in the German Clinical Trials Register (DRKS00015445). According to a statement by the Ethics Committee of the University Hospital and Medical Faculty Tübingen, in accordance with the German Federal Law § 3 Abs. 6 BDSG/LDSG BW, no formal ethics approval is required for the collection of anonymous data.

### 2.2 Survey instrument

No appropriate validated questionnaire could be found in literature to date. Therefore, we have developed a comprehensive questionnaire based on our research questions and adapted to the context of university hospitals. The questionnaire was pretested profoundly via the concurrent think aloud method (36) and additionally commented by *n* = 10 HCPs (nurses and physicians). After the pretest, the questionnaire was adapted accordingly. The survey was presented via Unipark software (Questback GmbH).

After a detailed introduction, covering the definition of CIM for this survey, the questionnaire contained three sections on content issues (“attitude”; “knowledge and need for information”; and “CIM at university hospitals”) and sociodemographic data. It included questions with an endpoint-labeled 10-point Likert scales, such as on attitude (10 = “very favorable”) and knowledge about CIM (10 = “very well”), matrix questions with a 4-point Likert scale on different general attitudes toward CIM at university hospitals and on specific therapies (4 = “I agree” or “useful” and response option “cannot judge”), and multiple-choice questions on interest in CIM training and factors influencing attitudes toward CIM. CIM use was asked in a dichotomous way. The therapy list of twenty CIM therapies included therapies from a preliminary study (patient survey (37)) and from various textbooks (38, 39). Examples for each therapy approach were given. Apart from open ended text questions and the category “other,” the selection of an answer in the questionnaire was required for proceeding to the next page (so no missing answers were allowed).

### 2.3 Recruitment of participants

Starting in July 2018, the survey link was separately sent out in sequence via the employee mailing lists of the four university hospitals in the federal state of Baden-Württemberg, Germany (Freiburg, Heidelberg, Ulm and Tübingen). This was followed by two mail reminders per site. The last e-mail was sent in September 2019. Since not every employee had their own personal business e-mail address at all four locations, in three locations internal house mail was additionally used. At one location, nurse department heads were contacted via e-mail and asked to distribute the link using a snowball approach.

### 2.4 Study population

The study aimed at a full survey of HCPs with direct patient contact at all four university hospitals in Baden-Württemberg with a focus on physicians and nurses.

### 2.5 Analysis

Only fully completed questionnaires including sociodemographic data (except age, years of work experience and specialty, which were voluntary information) were included in the analysis. In addition, questionnaires were excluded if the occupational group was not involved in patient care (e.g., “administration”) or if no occupational group was indicated. HCPs in training were not included. All 10-point Likert scales were scored as quasi-metric ordinal scales. IBM SPSS Statistics 28 was used for descriptive statistical analysis, subgroup analyses and comparisons between HCPs groups.

## 3 Results

### 3.1 Response rate

At location 1, about *n* = 5,500 professionals were contacted by e-mail. In the mailing list, staff was also included who was not the target group of the questionnaire (not involved in patient care). About *n* = 1,450 nurses and other HCPs were contacted via post. The response rate for all was about 5, and 8% for physicians.

At location 2, about *n* = 4,200 professionals were contacted by mailing list. The response rate of physicians was about 10%. As nurses and other HCPs did not have comprehensive professional mail accounts, further mail contacts (about *n* = 1,700) and the snowball principle were used, and for some departments (e.g., cardiology, psychosomatics) copies with the link were printed due to poor e-mail availability. For that reason, the response rate cannot be exactly determined.

At location 3, about *n* = 3,700 HCPs were contacted via e-mail and *n* = 2,800 nurses and other professionals were additionally contacted via house mail, from which max. *n* = 2,200 were contacted by both. The overall response rate for physicians was about 10%, for nurses about 14%, based on employee numbers.

At location 4, about *n* = 5,500 HCPs were contacted via e-mail and the response rate for all was about 13, 7% for physicians and 19% for nurses, and about 21% for others.

The overall response rate cannot be exactly determined.

### 3.2 Characteristics of the participants

Table 1 provides an overview of the sociodemographic data. A total of *n* = 2,026 HCPs participated, of which more than half were nurses and almost a third physicians, supplemented by other HCPs like physiotherapists, psychologists, midwives, and other professionals. More than two-thirds (70.9%, *n* = 1,437) of the participants were female and 14.1% (*n* = 286) reported additional training in CIM. The participants’ average age was 43.2 years (*SD* = 11.4), and they had 18.9 years of work experience on average (*SD =* 12.1).

**Table 1:** Sociodemographic data.

Demographic categories	*n*	%
Gender		
Female	1,437	70.9
Male	589	29.1
Profession		
Nurse	1,196	59.0
Physician	567	28.0
Physiotherapist	54	2.7
Psychotherapist/Psychologist	48	2.4
Midwife	37	1.8
Other	124	6.1
Leadership position		
Yes	417	20.6
No	1,609	79.4
Country of training		
Germany	1,960	96.7
Other	66	3.3
Additional training in CIM		
Yes	286	14.1
No	1,740	85.9
University hospital		
Location 1	735	36.3
Location 2	415	20.5
Location 3	550	27.1
Location 4	326	16.1
Total	2,026	100.0

Demographic categories with frequency (*n*) and valid percentage (%).

### 3.3 Attitude toward CIM

#### 3.3.1 General attitude toward CIM

The general attitude toward CIM (Question: “My general attitude toward Complementary and Integrative Medicine (CIM) would best be described as follows:”) tended to be clearly favorable (*M ± SD*: 7.43 ± 2.33; Likert scale: 1 = “very unfavorable,” 10 = “very favorable”). Midwives (9.05 ± 1.18), physiotherapists (8.44 ± 1.74), and nurses (8.08 ± 1.95) expressed the highest favorability, physicians (5.80 ± 2.39) the lowest. A Kruskal-Wallis test showed that attitudes toward CIM were influenced by professional group (*chi-square (2) =* 387.725, *p* < 0.001). Subsequent post-hoc tests (Dunn-Bonferroni tests) showed that physicians differed significantly from the other professional groups (except psychologists) (*z* = −7.872 to −18.630, *p* < 0.001).

The attitude toward CIM also differed significantly with gender: Male participants (6.20 ± 2.55) were more skeptical than female (7.93 ± 2.03) (Asymptotic Mann Whitney *U*: *z* = −14.334, *p* < 0.001, *r* = −0.318). Odds ratios in logistic regression have not been calculated due to the high degree of multicollinearity between profession and gender.

Participants who were not in a leadership position (7.57 ± 2.26) had a more favorable attitude than those who were (6.85 ± 2.51) (Asymptotic Mann Whitney *U*: *z* = −5,349, *p* < 0.001, *r* = −0.119).

For more details on general attitudes toward CIM, see Table 2.

**Table 2:** Healthcare professionals’ attitude toward CIM.

Attitude	Answer: “agree” or” rather agree” *n*, %				Answer “cannot judge” *n*, %
	All (*n* = 2,026)	Nurses (*n* = 1,196)	Physicians (*n* = 567)	Other (*n* = 263)	All (*n* = 2,026)
1. A holistic approach to patient care is important to me.	1,938, 95.7%	1,166, 97.5%	516, 90.0%	256, 97.3%	16, 0.8%
2. Patient expectations and values should be taken into consideration in treatment.	1,971, 97.3%	1,165, 97.4%	549, 96.8%	257, 97.7%	13, 0.6%
3. The placebo effect plays an important role in CIM.	1,266, 62.5%	618, 51.7%	501, 88.4%	147, 55.9%	289, 14.3%
4. The placebo effect plays an important role in conventional therapies.	1,017, 50.2%	527, 47.1%	358, 63.1%	132, 50.2%	243, 12.0%
5. The use of CIM has added value to patient care.	1,690, 83.4%	1,078, 90.1%	388, 68.4%	224, 85.2%	111, 5.5%
6. Physicians and nurses should distance themselves from CIM.	268, 13.2%	95, 7.9%	142, 25.0%	31, 11.8%	79, 3.9%
7. CIM contributes to patients’ health.	1,694, 83.6%	1,081, 90.4%	385, 67.9%	228, 86.7%	106, 5.2%
8. Patients are harmed in their health by CIM.	159, 7.8%	32, 2.7%	110, 19.4%	17, 6.5%	167, 8.2%
9. Patients are financially harmed by CIM.	537, 26.5%	167, 14.0%	308, 54.3%	62, 23.6%	327, 16.1%

HCPs’ rating for attitudes toward CIM (Question: “What is your opinion on the following statements?”) on a 4-point Likert scale (1 = “disagree,” 4 = “agree”) and answer option “cannot judge.” *n* = frequency, % = valid percentage. Professionals were divided into three categories.

#### 3.3.2 Attitude toward specific CIM therapies

Table 3 displays the attitudes toward specific CIM therapies in patient care. The therapies that were most frequently rated as useful or rather useful were relaxation therapy (e.g., progressive muscle relaxation, autogenic training) (*n* = 1,951; 96.3%), external applications (e.g., embrocations, wraps, pads) (*n* = 1,911; 94.3%), and massage (e.g., reflexology) (*n* = 1,836; 90.6%).

**Table 3:** Attitude toward CIM methods in patient care.

CIM method	Answer “useful” or “rather useful” *n*, %				Answer “cannot judge” *n*, %
	All (*n* = 2,026)	Nurses (*n* = 1,196)	Physicians (*n* = 567)	Other (*n* = 263)	All (*n* = 2,026)
Relaxation therapy (e.g., autogenic training, progressive muscle relaxation)	1,951, 96.3%	1,146, 95.8%	549, 96.8%	256, 97.3%	28, 1.4%
External applications (e.g., embrocations, wraps, pads)	1,911, 94.3%	1,172, 98.0%	489, 86.2%	250, 95.1%	25, 1.2%
Massage (e.g., reflexology)	1,836, 90.6%	1,145, 95.7%	441, 77.8%	250, 95.1%	40, 2.0%
Meditation/mindfulness	1,812, 89.4%	1,073, 89.7%	491, 86.6%	248, 94.3%	75, 3.7%
Meditative movement therapy (e.g., yoga, qigong, tai chi)	1,786, 88.2%	1,056, 88.3%	486, 85.7%	244, 92.8%	102, 5.0%
Acupuncture/acupressure	1,780, 87.9%	1,080, 90.3%	458, 80.8%	242, 92.0%	81, 4.0%
Manual medicine (e.g., chiropractic, osteopathy, cranio-sacral therapy)	1,703, 84.1%	1,067, 89.2%	400, 70.5%	236, 89.7%	112, 5.5%
Hydrotherapy/balneotherapy (e.g., Kneipp, alternating showers, steam bath)	1,612, 79.6%	972, 81.3%	422, 74.4%	218, 82.9%	170, 8.4%
Phytotherapy/herbal medicine	1,533, 75.7%	946, 79.1%	380, 67.0%	207, 78.7%	233, 11.5%
Nutritional therapy (e.g., special diets, fasting)	1,531, 75.6%	912, 76.3%	400, 70.5%	219, 83.3%	148, 7.3%
Aromatherapy	1,304, 64.4%	958, 80.1%	185, 32.6%	161, 61.2%	236, 11.6%
Homeopathy	1,191, 58.8%	888, 74.2%	119, 21.0%	184, 70.0%	116, 5.7%
Nutritional supplements (e.g., vitamins, minerals, trace elements)	1,076, 53.1%	688, 57.5%	235, 41.4%	153, 58.2%	227, 11.2%
Microbiotic therapy (e.g., probiotics)	1,009, 49.8%	593, 49.6%	296, 52.2%	120, 46.6%	102, 5.0%
Ayurvedic medicine	965, 47.6%	653, 54.6%	173, 30.5%	139, 52.9%	619, 30.6%
Anthroposophic medicine	905, 44.7%	653, 54.6%	127, 22.4%	125, 47.5%	513, 25.3%
Drainage therapy (e.g., leech therapy, cupping)	813, 40.1%	568, 47.5%	115, 20.3%	130, 49.4%	489, 24.1%
Neural therapy (e.g., wheal therapy)	697, 34.4%	465, 38.9%	132, 23.3%	100, 38.0%	759, 37.5%
Mistletoe therapy	665, 32.8%	463, 38.7%	121, 21.3%	81, 30.8%	762, 37.6%
Other^a^	111, 5.5%	76, 6.4%	14, 2.5%	21, 8.0%	194, 9.6%

HCPs’ rating for specific CIM methods (Question: “In general, how useful do you rate the following CIM therapies for patients?”) on a 4-point Likert scale (1 = “not useful,” 4 = “useful”) and answer option “cannot judge.” *n* = frequency, % = valid percentage. Sorted by *n* (all) for answer “useful” or “rather useful.” HCPs were divided into three categories. ^a^*n* = 321 (due to missing data).

#### 3.3.3 Attitude toward CIM as task of university hospitals

For attitudes toward CIM at university hospitals see Table 4. Most of the participants agreed or rather agreed that providing CIM to patients (*n* = 1,408; 69.5%), as well as research on (*n* = 1,763; 87.0%) and counseling (*n* = 1,601; 79.0%) about CIM are tasks of university hospitals. Physicians and nurses differed significantly in their attitude toward providing CIM to patients (asymptotic Mann Whitney *U*: *z* = −16.049, *p* < 0.001), counseling about CIM (*z* = −10.046, *p* < 0.001), and research on CIM (*z* = −5.304, *p* < 0.001). Additionally, physicians and nurses showed significant differences regarding interprofessional care for CIM (*z* = −12.948, *p* < 0.001).

**Table 4:** CIM as task of university hospitals.

Attitude	Answer “agree” or “rather agree” *n*, %				Answer “cannot judge” *n*, %
	All (*n* = 2,026)	Nurses (*n* = 1,196)	Physicians (*n* = 567)	Other (*n* = 263)	All (*n* = 2,026)
1. Counseling about CIM is one of the tasks of university hospitals.	1,601, 79.0%	1,003, 83.9%	383, 67.5%	215, 81,7%	84, 4,1%
2. Providing CIM to patients is one of the tasks of university hospitals.	1,408, 69.5%	956, 79.9%	262, 46.2%	190, 72.2%	93, 4.6%
3. Research on CIM is one of the tasks of university hospitals.	1,763, 87.0%	1,062, 88.8%	472, 83.2%	229, 87.1%	66, 3.3%
4. An outpatient clinic for CIM at university hospitals. is desirable.	1,442, 71.2%	962, 80.4%	279, 49.2%	201, 76.4%	103, 5,1%
5. A consulting service for CIM at university hospitals is desirable.	1,478, 73.0%	989, 82.7%	294, 51.9%	195, 74.1%	105, 5.2%
6. CIM should be an interprofessional task at university hospitals.	1,588, 78.4%	1,020, 85.3%	356, 62.8%	212, 80.6%	143, 7.1%

HCPs’ attitudes toward CIM as task of university hospitals (Question: “What is your opinion on the following statements about CIM at university hospitals?”) on a 4-point Likert scale (1 = “disagree,” 4 = “agree”) and answer “cannot judge.” *n* = frequency, % = valid percentage. HCPs are divided in three categories.

### 3.4 CIM use at university hospitals

41.7% of the participants involved CIM in patient care (Question: “Do you use CIM therapies with patients in your clinical practice?”). The highest use was shown by midwives (86.5%, *n* = 32) and physiotherapists (79.6%, *n* = 43). The lowest use had physicians (33.2%, *n* = 188).

### 3.5 Knowledge and communication about CIM

The personal level of knowledge about CIM (Question: “How well do you feel informed about CIM overall?”) was assessed as rather average (*M ± SD*: 5.83 ± 2.03; scale: 1 = “very poorly,” 10 = “very well”). Midwives reported the highest level of knowledge (7.24 ± 1.95), physicians the lowest (5.54 ± 2.02).

Less than one third (*n* = 578, 28.5%) of participants agreed or rather agreed that they feel confident in counseling patients about CIM and less than half of the participants (*n* = 859; 42.4%) agreed or rather agreed that they are often asked about CIM by patients. For comparison between professional groups see Table 5.

**Table 5:** Communication with university hospital patients about CIM.

Attitude	Answer “agree” or” rather agree” *n*, %				Answer “cannot judge” *n*, %
	All (*n* = 2,026)	Nurses (*n* = 1,196)	Physicians (*n* = 567)	Other (*n* = 263)	All (*n* = 2,026)
1. From my point of view, patient interest in CIM has increased in the last few years.	1,553, 76.7%	905, 75.7%	441, 77.8%	207, 78.7%	212, 10.5%
2. Patients often ask me about CIM topics.	859, 42.4%	453, 37.9%	266, 46.9%	140, 53.2%	87, 4.3%
3. I often actively ask my patients about their need for or use of CIM.	542, 26.8%	328, 27.4%	117, 20.6%	97, 36.9%	91, 4.5%
4. I feel confident in counseling patients about CIM.	578, 28.5%	284, 23.7%	187, 33.0%	107, 40.7%	129, 6.4%

HCPs’ attitudes toward communication about CIM (Question: “What is your opinion on the following statements about the role of CIM in your interaction with patients?”) on a 4-point Likert scale (1 = “disagree,” 4 = “agree”) and answer “cannot judge.” *n* = frequency, % = valid percentage. HCPs are divided in three categories.

#### 3.5.1 Education about CIM – past and future training

The vast majority of participants (*n* = 1,764, 87.1%) agreed or rather agreed that CIM training at university hospitals is important to them. The importance attributed to CIM in further training (Question: “How important was CIM in your previous training and further training?”) was rated as rather low (*M ± SD*:3.55 ± 2.21; scale: 1 = “not at all important,” 10 = “very important”). Midwives saw the highest (7.11 ± 2.41), and physicians the lowest (3.08 ± 1.84) importance.

The most frequently requested topics for training (Question: “For which of the following CIM therapies do you have an interest in further information (e.g., in the form of training courses)?”) in a multiple choice question (*n* = 2,026) were acupuncture/acupressure (*n* = 1,025), relaxation therapy (*n* = 984), manual medicine (e.g., chiropractic, osteopathy, cranio-sacral therapy) (*n* = 870), external applications (e.g., embrocations, wraps, pads) (*n* = 829), and meditation/mindfulness (*n* = 806).

## 4 Discussion

To the best of our knowledge, this cross-sectional study about attitudes toward, knowledge about and interest in CIM training is the first multicenter full survey at university hospitals in the German-speaking area. The aim of the present study was to investigate the field of CIM at university hospitals within a multicenter study in Germany.

The general attitude toward CIM showed a clear positive trend for all participants (*M ± SD*: 7.43 ± 2.33), which varied across the professional groups. Our results are consistent with other surveys in the German-speaking area by Trimborn et al. (28) and Aveni et al. (34). The academic centers in Baden-Württemberg revealed that nurses had a more positive attitude compared to physicians. Our results showed that midwifes and physiotherapists are even more favorable toward CIM than nurses. Midwives’ support for CIM was already explored in several studies (24, 40). Possible reasons for the discrepancy between physicians and other professionals may be differences in education and practice. While acupuncture, massage, and relaxation techniques are a part of classic midwifery textbooks (41), complementary medicine is often taught separately and during post-graduate education to physicians. Consistent with our results, physiotherapists in Sweden recommended CIM more than physicians and nurses according to Bjerså et al. (42). Our findings again provide support for gender differences in attitudes (28, 43).

Another aim was to examine the attitudes toward specific CIM therapies. Professional respondents in our study found relaxation therapy, external applications, and massage the most useful for patient care. For most of the therapies rated as useful by HCPs, positive effects have been shown in studies, such as reduction of chronic pain (6), or treatment-related symptoms during chemotherapy (44) by *relaxation therapy* or reduction of anxiety and depression (45) by *massage therapy*. For the field of *external applications,* a recent article by Stolz et al. underlined a great potential for independent use by patients (46). Considering the high interest of HCPs in CIM, Mühlenpfordt et al. have underlined a high demand for more future research in this field (47). According to Aveni et al. personal experience is a significant factor for HCPs at a university hospital in Switzerland when forming their opinions on CIM (34). Positive experience with external applications in patient care might be a reason for our results. Given the cost-effectiveness for wraps, etc., this could be another reason for pragmatic approach in patient care.

In a survey among university hospital patients in Germany by Lederer et al., exercise, herbal medication, and dietary supplements were the three most used CIM methods (15). The effectiveness of some *phytotherapeutic* approaches has been demonstrated in several randomized controlled trials (e.g., turmeric in patients with irritable bowel syndrome (48)) and systematic reviews (e.g., cranberries for prevention of urinary infections (7), St. John’s wort for moderate depression (49), and psyllium for chronic constipation (9)). In our study, HCPs did not highlight phytotherapy as useful. A possible reason for this difference in prioritization could be the setting: In the primary care setting, CIM (e.g., over-the-counter-phytotherapy) is often used for self-limiting diseases (50).

For the first time in Germany, the focus on the university hospital setting especially and the attitudes of HCPs working in this setting was explored in our study. Participants showed a distinct positive attitude toward CIM integration at university hospitals. Overall, 80% of all participants and about two-thirds of the physicians agreed or rather agreed that counseling about CIM is a task of university hospitals. According to our study, more than 40% of the participants were already using CIM in patient care. Midwives, physiotherapists, and psychologists showed higher rates of use than nurses in our study. That can possibly be explained by the fact that for physiotherapists, manual therapies are often integrated into their daily work, whereas psychologists are specifically trained in relaxation therapies. In line with other studies, less than half of the participants regularly communicated about CIM with their patients (51). To close this gap, HCPs communicative skills should be trained, especially to address patients’ needs. When it comes to CIM in the university hospital setting, the highest acceptance was shown for research: More than 80% of the participants agreed or rather agreed that CIM research is a university hospitals’ task. To meet this demand, an expansion of university research centers could be helpful, as in Germany, out of 38 university hospitals, only 13 operate an outpatient clinic for CIM and six have an endowed chair or professorship for CIM research (partly the same) (52). The WHO Global Report on Traditional and Complementary Medicine 2019 highlights the absence of national funding, national expert committees, a national agenda, and a national research institute for CIM in most European countries (Germany included) (53). Given the positive attitude to CIM and the involvement in clinical practice at university hospitals according to our study, these may be structural gaps that need to be filled.

Participants in our study supported an interprofessional approach for CIM at university hospitals. More than 70% agreed or rather agreed that CIM at university hospitals should be an interprofessional task, with nurses more likely to agree than physicians. The high demand for interprofessionality was also expressed in a study by Homberg and Stock-Schröer, who used a qualitative approach to investigate the advantages and disadvantages of interprofessional CIM education in Germany and Switzerland (54). They concluded that an interprofessional approach could help to overcome stereotypes. Prill et al. conducted a mixed method study about interprofessional teaching regarding CIM and their participants emphasized the relevance of team meetings as a factor promoting interprofessional collaboration (55). This could also be relevant for university hospitals.

### 4.1 Knowledge about CIM

Knowledge about CIM was another focus of our study. The self-assessed knowledge (information level) about CIM tended to be average (*M ± SD*: 5.83 ± 2.03), and only less than one third of participants (28.5%) agreed or rather agreed that they felt competent enough to counsel patients about CIM. Comparison with other studies is rather difficult because the questions were asked differently. In line with several other surveys (24, 27, 34), a lack of knowledge was also mentioned. The discrepancy between positive attitude and a subjective average level of knowledge that was shown in our study, was also found among nurses in the Chang and Chang survey (24). Several other studies cited a lack of knowledge as a main reason for not discussing CIM therapies with patients (27, 51). This may have contributed to the fact that less than 30% of HCPs in our study felt confident in counseling patients about CIM. To address this issue, high-quality evidence-based education on CIM should be provided to professionals, ideally in their training period as participants rated the importance of CIM in their previous education as rather low. For structured training, the definition of competencies to be acquired could also be helpful. For physician training in general practice on CIM, Valentini et al. developed a competency catalog for Germany with a multi-level, peer-based approach (56). This catalog could serve as a basis for other professions.

### 4.2 Interest in training about CIM

The participants expressed a high interest in training about CIM. More than 80% of the participants agreed or rather agreed that training in CIM at university hospitals is important to them. The top three topics for additional training mentioned in our study were acupuncture/acupressure, relaxation therapy, and manual medicine. With regard to acupuncture, a high popularity among the patients in Germany (57) is known, and the costs are covered by public health insurance for some indications (58). Furthermore, in Germany, physicians can take structured additional qualifications in acupuncture which are awarded by the medical association (59). Interestingly, acupuncture/acupressure was among the most requested therapies for training at university hospitals, even if it was not among the most useful therapies for patient care. Possible reasons for this high interest on training in acupuncture might be due to the robust body of evidence on acupuncture with, for example, over 2,100 positive recommendations for acupuncture in clinical guidelines for over 200 indications (60).

### 4.3 Strengths and limitations

To the best of our knowledge, this is the first multicenter study of CIM at university hospitals. We aimed for a full survey study and over *n* = 2,000 participants took part in the survey. Therefore, subgroup analyses and comparisons between HCPs groups were possible. Another strength is the comprehensive questionnaire with a detailed list of different CIM methods and examples to ensure a consistent understanding of terms. The web-based and anonymous research design minimized social desirability as a potential source of bias. The tendency toward the middle as a possible bias was avoided using straight scales.

Nevertheless, the results should be interpreted with caution due to some important limitations of our study that need to be considered. First, we did not validate our questionnaire. To the best of our knowledge, no appropriate validated questionnaire has been published. We therefore discussed the questionnaire in an interprofessional team, conducted pretests and tried to explain the terms we used with examples to minimalize comprehension problems. Another limitation of this study is that the process of recruiting participants varied across study sites and therefore the response rate of the study cannot be accurately determined. Furthermore, the response rate across the different locations did not exceed 20 percent at any location or within any profession. Overall, the response rate was lower than in a similar study by Aveni et al., where the response rate was approximately 25% (34). In contrast to our study, e-mail accessibility in university hospitals may be better in Switzerland. It is difficult to draw a balanced picture here and it is to assume that especially HCPs with a very positive attitude regarding CIM have responded to the questionnaire and are overrepresented here. Nonetheless, as the area of CIM is very polarizing in general, it is also to assume that also very skeptical HCPs felt called to take part, especially within the profession of physicians.

Furthermore, it is possible that the link to the survey was distributed beyond the university hospitals since there was no personalized access. To reduce a potential selection bias, the survey invitation mail was formulated as neutrally as possible with evidence-based examples like acupuncture and phytotherapy. Unclear definitions of different therapies included make it difficult in some cases to compare studies. Nevertheless, similar trends can be identified. Also, the transferability of the results to other regions in Germany and internationally should be discussed.

The reasons for participants’ attitudes toward CIM were not considered in this study. Supplementary qualitative research may be helpful to address this issue. In addition, barriers to the use of CIM at university hospitals should be addressed in future research.

### 4.4 Conclusion

The present study emphasizes the pronounced interest of HCPs in CIM within the context of university hospitals in Germany. Despite the interest, the moderate level of CIM knowledge among HCPs coupled with the limited emphasis of CIM within continuing education frameworks, underscores a need for enhanced CIM training. This becomes even more evident for physicians, who were most skeptical and reported the lowest knowledge. Notably, over 40% of the HCPs incorporate CIM into their clinical practice at university hospitals, yet a smaller proportion felt able to discuss CIM competently with patients. Our study elucidates the widespread utilization of CIM by HCPs in a university hospital setting, showing the substantial demand for an evidence-based interprofessional approach to CIM. Finally, our findings indicate a high level of interest among HCPs for comprehensive training in CIM– a component that warrants integration into medical education curricula. To optimize patient care and patient safety, it is essential to identify and integrate CIM modalities with robust scientific evidence, spanning from clinical application to patient communication.

## Data availability statement

The raw data supporting the conclusions of this article will be made available by the authors, without undue reservation.

## Ethics statement

The requirement of ethical approval was waived by Ethics Committee of the University Hospital and Faculty of Medicine Tübingen. The studies were conducted in accordance with the local legislation and institutional requirements. The participants provided their written informed consent to participate in this study.

## Author contributions

DH: Writing – original draft, Writing – review & editing, Conceptualization, Data curation, Formal analysis, Investigation, Project administration, Resources, Visualization. CK: Writing – original draft, Writing – review & editing, Data curation, Formal analysis, Methodology, Project administration, Visualization. RS: Writing – original draft, Writing – review & editing, Conceptualization, Methodology. RH: Writing – original draft, Writing – review & editing, Conceptualization, Funding acquisition, Investigation, Project administration, Resources, Supervision. YS: Writing – original draft, Writing – review & editing, Conceptualization, Funding acquisition, Investigation, Project administration, Resources, Supervision. KH: Writing – original draft, Writing – review & editing, Conceptualization, Investigation, Project administration, Resources, Supervision. ThS: Writing – original draft, Writing – review & editing, Conceptualization, Funding acquisition, Investigation, Project administration, Resources, Supervision. TaS: Writing – original draft, Writing – review & editing, Conceptualization, Funding acquisition, Investigation, Project administration, Resources, Supervision. SJ: Writing – original draft, Writing – review & editing, Conceptualization, Funding acquisition, Investigation, Methodology, Project administration, Resources, Supervision. JV: Writing – original draft, Writing – review & editing, Conceptualization, Data curation, Formal analysis, Funding acquisition, Investigation, Methodology, Project administration, Resources, Supervision, Visualization.
