# Supplementary material for: Exploring the gap: attitudes, knowledge, and training needs in complementary and integrative medicine among healthcare professionals at German university hospitals
Source: Front Med (Lausanne). 2024 May 9;11:1408653. doi: 10.3389/fmed.2024.1408653 (PMC11111851; doi:10.3389/fmed.2024.1408653)
Supplement: Supplementary file 1 [file Data_Sheet_1.pdf]

*Supplementary Material*

# Questionnaire- English Translation

## Introduction

## Information About the Online Survey on Complementary and Natural Health Medical Procedures

**Who can participate in the survey?**
The survey is addressed to **all employees with patient contact at the University Hospitals of Freiburg, Heidelberg, Tübingen and Ulm**, regardless of their profession or function (physicians, nurses, etc.).

**What is the survey about?**
The demand for **complementary and natural medicine methods** (e.g. herbal remedies, acupuncture, aromatherapy, yoga, etc.) in the population is high. Some of these methods are already finding their way into conventional patient care. For this reason, one increasingly speaks of **"complementary and integrative medicine" (CIM)**, which is understood as an evidence-based integration of the above-mentioned methods. With this survey we would like to find out about **your experiences, opinions and information** needs regarding CIM. Completing the questionnaire will take **approx. 7 - 10 minutes**.

**Who is conducting the study?**
The study is conducted within the framework of the **Academic Center for Complementary and Integrative Medicine (AZKIM)** and is funded by the Ministry of Science, Research and Art Baden-Württemberg. (www.azkim.de).

**What happens to your information?**
The data is collected anonymously, so that it is not possible to trace it back to you personally. With the completion of this questionnaire, you agree to having the anonymously collected data evaluated and published.

**More questions?**
Please feel free to contact us.

- Cand. med. Daniela Hesmert, MD candidate (daniela.hesmert@med.uni-tuebingen.de)
- Prof. Dr. med. Stefanie Joos, Medical Director, Institute for General Practice and und Interprofessional Care, Tübingen University Clinic (stefanie.joos@med.uni-tuebingen.de)


**Thank you so much...**
... in advance for your support!

UNIVERSITÄTS KLINIKUM FREIBURG UniversitätsKlinikum Heidelberg Universitätsklinikum Ulm UNIVERSITÄTS KLINIKUM TÜBINGEN

**Attitudes- page 1**

Akademisches
Zentrum
Komplementäre &
Integrative
Medizin

# 1. Attitudes

**1.1 CIM is a term used to describe complementary medicine and natural health practices.** ?
**My general attitude toward Complementary and Integrative Medicine (CIM) would best be described as follows:**

*(Please use the slider to set the appropriate value. You can see examples by clicking on the green question mark.)*

very unfavorable | very favorable

|______|______|______|______|______|______|______|______|______|

*[Clicking the green question mark displays the following text:*

*„Examples for CIM therapies:*

*acupuncture/acupressure, anthroposophic medicine, aromatherapy, drainage therapy (e.g. leech therapy, cupping), external applications (e.g. embrocations, wraps, pads), ayurvedic medicine, relaxation therapies (e.g. autogenic training, progressive muscle relaxation), nutritional therapy (e.g. special diets, fasting), homeopathy, hydrotherapy/balneotherapy (e.g. Kneipp, alternating showers, steam bath), manual medicine (e.g. chiropractic, osteopathy, cranio-sacral therapy), massage (e.g. reflexology), meditation/mindfulness, meditative movement therapy (e.g. yoga, qigong, tai chi), microbiotic therapy (e.g. probiotics), mistletoe therapy, nutritional supplements (e.g. vitamins, minerals, trace elements), neural therapy (e.g.wheal therapy), phytotherapy/herbal medicine"]*

**1.2 What is your opinion on the following statements?**

*(Please select an answer in each row)*

| | disagree | rather disagree | rather agree | agree | **cannot judge** |
|---|---|---|---|---|---|
| A holistic approach to patient care is important to me. | ☐ | ☐ | ☐ | ☐ | ☐ |
| Patient expectations and values should be taken into consideration in treatment. | ☐ | ☐ | ☐ | ☐ | ☐ |
| The placebo effect plays an important role in CIM. | ☐ | ☐ | ☐ | ☐ | ☐ |

| | | | | | |
|---|---|---|---|---|---|
| The placebo effect plays an important role in conventional therapies. | ☐ | ☐ | ☐ | ☐ | ☐ |
| The use of CIM has added value to patient care. | ☐ | ☐ | ☐ | ☐ | ☐ |
| Physicians and nurses should distance themselves from CIM. | ☐ | ☐ | ☐ | ☐ | ☐ |
| CIM contributes to patients' health. | ☐ | ☐ | ☐ | ☐ | ☐ |
| Patients are harmed in their health by CIM. | ☐ | ☐ | ☐ | ☐ | ☐ |
| Patients are financially harmed by CIM. | ☐ | ☐ | ☐ | ☐ | ☐ |

Akademisches
Zentrum
Komplementäre &
Integrative
Medizin

## 1.3 In general, how useful do you find the following CIM therapies for patients?

*(Please select an answer in each row)*

| | not useful | rather not useful | rather useful | useful | cannot judge |
|---|---|---|---|---|---|
| acupuncture/acupressure | ☐ | ☐ | ☐ | ☐ | ☐ |
| anthroposophic medicine | ☐ | ☐ | ☐ | ☐ | ☐ |
| aromatherapy | ☐ | ☐ | ☐ | ☐ | ☐ |
| drainage therapy *(e.g. leech therapy, cupping)* | ☐ | ☐ | ☐ | ☐ | ☐ |
| external applications *(e.g. embrocations, wraps, pads)* | ☐ | ☐ | ☐ | ☐ | ☐ |
| ayurvedic medicine | ☐ | ☐ | ☐ | ☐ | ☐ |
| relaxation therapies *(e.g. progressive muscle relaxation, autogenic training)* | ☐ | ☐ | ☐ | ☐ | ☐ |
| nutritional therapy *(e.g. special diets,fasting)* | ☐ | ☐ | ☐ | ☐ | ☐ |
| homeopathy | ☐ | ☐ | ☐ | ☐ | ☐ |
| hydrotherapy/balneotherapy *(e.g. Kneipp, alternating showers, steam bath)* | ☐ | ☐ | ☐ | ☐ | ☐ |
| manual medicine *(e.g. chiropractic, osteopathy, cranio-sacral therapy)* | ☐ | ☐ | ☐ | ☐ | ☐ |
| massage *(e.g. reflexology)* | ☐ | ☐ | ☐ | ☐ | ☐ |
| meditation/mindfulness | ☐ | ☐ | ☐ | ☐ | ☐ |
| meditative movement therapy *(e.g. yoga, qigong, tai chi)* | ☐ | ☐ | ☐ | ☐ | ☐ |
| microbiotic therapy *(e.g. probiotics)* | ☐ | ☐ | ☐ | ☐ | ☐ |
| mistletoe therapy | ☐ | ☐ | ☐ | ☐ | ☐ |
| nutritional supplements *(e.g. vitamins, minerals, trace elements)* | ☐ | ☐ | ☐ | ☐ | ☐ |
| neural therapy *(e.g. wheal therapy)* | ☐ | ☐ | ☐ | ☐ | ☐ |
| phytotherapy/herbal medicine | ☐ | ☐ | ☐ | ☐ | ☐ |
| other | ☐ | ☐ | ☐ | ☐ | ☐ |

**Attitudes -page 3**

Akademisches
Zentrum
Komplementäre &
Integrative
Medizin

## 1.4 What most influences your opinion of CIM?

*(maximum 3 choices)*

| | |
|---|---|
| ☐ | guidelines |
| ☐ | personal experience |
| ☐ | Internet |
| ☐ | professional literature *(e.g., studies, journals)* |
| ☐ | feedback from patients |
| ☐ | education/training |
| ☐ | exchanging ideas with colleages |
| ☐ | television, newspaper articles |
| ☐ | other:_________ |

?

## 1.5 Have you ever used CIM therapies for yourself?

*(You can see examples by clicking on the green question mark.)*

| | |
|---|---|
| ☐ | yes |
| ☐ | no |

## 1.5.1 If yes, which CIM therapies and for what reason?

**Knowledge-page 1**

Akademisches
Zentrum
Komplementäre &
Integrative
Medizin

# 2. Knowledge and information needs

## 2.1 How well informed do you feel about CIM overall?

*(Please use the slider to set the appropriate value.)*

very poorly | very well

## 2.2 How important has CIM been in your education and training to date?

*(Bitte wählen Sie mit dem Schieberegler den entsprechenden Wert aus.)*
*(Please use the slider to set the appropriate value.)*

not at all important | very important

## Knowledge- page 2

Akademisches
Zentrum
Komplementäre &
Integrative
Medizin

### 2.3 For which of the following CIM therapies are you interested in further information (e.g. in the form of training courses)?

*(Select all that apply)*

| | |
|---|---|
| **I am not interested in any of these therapies.** | ☐ |
| acupuncture/acupressure | ☐ |
| anthroposophic medicine | ☐ |
| aromatherapy | ☐ |
| drainage therapy *(e.g. leech therapy, cupping)* | ☐ |
| external applications *(e.g. embrocations, wraps, pads)* | ☐ |
| ayurvedic medicine | ☐ |
| relaxation therapies *(e.g. progressive muscle relaxation, autogenic training)* | ☐ |
| nutritional therapy *(e.g. special diets,fasting)* | ☐ |
| homeopathy | ☐ |
| hydrotherapy/balneotherapy *(e.g. Kneipp, alternating showers, steam bath)* | ☐ |
| manual medicine *(e.g. chiropractic, osteopathy, cranio-sacral therapy)* | ☐ |
| massage *(e.g. reflexology)* | ☐ |
| meditation/mindfulness | ☐ |
| meditative movement therapy *(e.g. yoga, qigong, tai ji)* | ☐ |
| microbiotic therapy *(e.g. probiotics)* | ☐ |
| mistletoe therapy | ☐ |
| nutritional supplements *(e.g. vitamins, minerals, trace elements)* | ☐ |
| neural therapy *(e.g. wheal therapy)* | ☐ |
| phytotherapy/herbal medicine | ☐ |
| other | ☐ |

Akademisches
Zentrum
Komplementäre &
Integrative
Medizin

# 3. CIM at university hospitals

### 3.1 Do you use CIM therapies with patients in your clinical practice?

?

*(You can see examples by clicking on the green question mark.)*

| | |
|---|---|
| ☐ | yes |
| ☐ | no |

### 3.1.1 If yes, which CIM therapies and for what reason?

### 3.2 What is your opinion on the following statements about the role of CIM in your interaction with patients?

*(Please select an answer in each row)*

| | disagree | rather disagree | rather agree | agree | **cannot judge** |
|---|---|---|---|---|---|
| From my point of view, patient interest in CIM has increased in the last few years. | ☐ | ☐ | ☐ | ☐ | ☐ |
| Patients often ask me about CIM topics. | ☐ | ☐ | ☐ | ☐ | ☐ |
| I often actively ask my patients about their need for or use of CIM. | ☐ | ☐ | ☐ | ☐ | ☐ |
| I feel confident in advising patients about CIM. | ☐ | ☐ | ☐ | ☐ | ☐ |

Akademisches
Zentrum
Komplementäre &
Integrative
Medizin

## 3.3 What is your opinion on the following statements about CIM at university hospitals?

*(Please select an answer in each row)*

| | disagree | rather disagree | rather agree | agree | **cannot judge** |
|---|---|---|---|---|---|
| Counseling about CIM is one of the tasks of university hospitals. . | ☐ | ☐ | ☐ | ☐ | ☐ |
| Providing CIM to patients is one of the tasks of university hospitals. | ☐ | ☐ | ☐ | ☐ | ☐ |
| Research on CIM is one of the tasks of university hospitals | ☐ | ☐ | ☐ | ☐ | ☐ |
| I think training in CIM at university hospitals is important. | ☐ | ☐ | ☐ | ☐ | ☐ |
| An outpatient clinic for CIM at university hospitals is desirable. | ☐ | ☐ | ☐ | ☐ | ☐ |
| A consulting service for CIM at university hospitals is desirable. | ☐ | ☐ | ☐ | ☐ | ☐ |
| CIM should be an interprofessional task at university hospitals. | ☐ | ☐ | ☐ | ☐ | ☐ |
| I think the attitude of my colleagues and superiors towards CIM is fairly positive. | ☐ | ☐ | ☐ | ☐ | ☐ |

**Sociodemographics-page 1**

Akademisches
Zentrum
Komplementäre &
Integrative
Medizin

# 4. Sociodemogaphics

## 4.1 Your age

*(in years):*

_ _

## 4.2 Your gender:

| | |
|---|---|
| ☐ | male |
| ☐ | female |

## 4.3 Your professional qualification:

| | |
|---|---|
| **Physicians:** | |
| ☐ | resident |
| ☐ | specialist |
| **Nurses:** | |
| ☐ | nursing assistant |
| ☐ | registered nurse |
| ☐ | specialized nurse |
| **Other professions (e. g., physiotherapy)** | |
| ☐ | ___________________________________ |

## 4.4. Your work experience

*(in years)*

_ _

## 4.5 Do you have completed qualifications in CIM?

| | |
|---|---|
| ☐ | yes |
| ☐ | no |

## 4.5.1 If yes, which?

_______________________________________________

_______________________________________________

**4.6 Country where you received most of your medical/nursing education:**

| | |
|---|---|
| ☐ | Germany |
| ☐ | Other:_________________ |

**4.7 Department in which you currently work:**

*(e. g., gynecology, neurosurgery)*

_______________________________________________________________________________

_______________________________________________________________________________

**4.8 Do you have a leadership position?**

| | |
|---|---|
| ☐ | yes |
| ☐ | no |

**4.9 Your amount of employment time:**

| | |
|---|---|
| ☐ | under 30% |
| ☐ | 30-70% |
| ☐ | 70-100% |

**4.10 Location of university hospital:**

| | |
|---|---|
| ☐ | Freiburg |
| ☐ | Heidelberg |
| ☐ | Tübingen |
| ☐ | Ulm |

**Comments-page 1**

Akademisches
Zentrum
Komplementäre &
Integrative
Medizin

# 5. Comments

### 5.1 Do you have any other comments about CIM?

### 5.2 Do you have any comments about this study and this questionnaire?

We appreciate your feedback!

**Endseite/ Last page**

Akademisches
Zentrum
Komplementäre &
Integrative
Medizin

**Thank you for participating in this study!**
Your information has been saved. You can now close this window.

**Do you have any questions, or would you like to be informed about the results of the survey?**
You can feel free to contact us.


- Cand. med. Daniela Hesmert, MD candidate (daniela.hesmert@med.uni-tuebingen.de)
- Prof. Dr. med. Stefanie Joos, Medical Director, Institute for General Practice and und Interprofessional Care, Tübingen University Clinic (stefanie.joos@med.uni-tuebingen.de)


UNIVERSITÄTS KLINIKUM FREIBURG UniversitätsKlinikum Heidelberg Universitätsklinikum Ulm UNIVERSITÄTS KLINIKUM TÜBINGEN
